# Integration of 3D nuclear imaging in 3D mapping system for ventricular tachycardia ablation in patients with implanted devices: Perfusion/voltage retrospective assessment of scar location

**DOI:** 10.1016/j.hroo.2022.06.008

**Published:** 2022-06-27

**Authors:** Bernard Thibault, Louis-Philippe Richer, Luke C. McSpadden, Kyungmoo Ryu, Martin Aguilar, Julia Cadrin-Tourigny, Rafik Tadros, Blandine Mondésert, Léna Rivard, Katia Dyrda, Marc Dubuc, Laurent Macle, Mario Talajic, Paul Khairy, Peter G. Guerra, Denis Roy, Jean Grégoire, François Harel

**Affiliations:** ∗ Electrophysiology Service, Montreal Heart Institute, Université de Montréal, Montreal, Canada; † Nuclear Medicine Department, Montreal Heart Institute, Université de Montréal, Montreal, Canada; ‡ Abbott, Sylmar, California

**Keywords:** Implanted device, Ischemic scar, Perfusion imaging, SPECT/CT, Voltage mapping, Ventricular tachycardia, Ventricular ischemia

## Abstract

**Background:**

The identification of low-voltage proarrhythmic areas for catheter ablation of scar-mediated ventricular tachycardia (VT) remains challenging. Integration of myocardial perfusion imaging (single-photon emission computed tomography/computed tomography; SPECT/CT) and electroanatomical mapping (EAM) may improve delineation of the arrhythmogenic substrate.

**Objective:**

To assess the feasibility of SPECT/CT image integration with voltage maps using the EnSite Precision system (Abbott) in patients undergoing scar-mediated VT ablation.

**Methods:**

Patients underwent SPECT/CT imaging prior to left ventricular (LV) EAM with the EnSite Precision mapping system. The SPECT/CT, EAM data, and ablation lesions were retrospectively co-registered in the EnSite Precision system and exported for analysis. Segmental tissue viability scores were calculated based on SPECT/CT perfusion and electrogram bipolar voltage amplitude. Concordance, specificity, and sensitivity between the 2 modalities as well as the impact of SPECT/CT spatial resolution were evaluated.

**Results:**

Twenty subjects (95% male, 67 ± 7 years old, left ventricular ejection fraction 36% ± 11%) underwent EAM and SPECT/CT integration. A concordance of 70% was found between EAM and SPECT/CT for identification of cardiac segments as scar vs viable, with EAM showing a 68.5% sensitivity and 76.4% specificity when using SPECT/CT as a gold standard. Projection on low-resolution 3D geometries led to an average decrease of 38% ± 22% of the voltage points used.

**Conclusion:**

The study demonstrated the feasibility of integrating SPECT/CT with EAM performed retrospectively for characterization of anatomical substrates during VT ablation procedures.

**Key Findings**

▪ The study demonstrated, using an integration developed retrospectively, the feasibility of integrating single-photon emission computed tomography/computed tomography with impedance-based electroanatomical mapping for characterization of anatomical substrates during ventricular tachycardia ablation procedures.
▪ The discrepancy between the spatial resolution of a 3D geometry derived from an image modality and number of voltage points acquired during electroanatomical mapping can impact the final voltage map projected on the 3D geometry.
▪ Future work will investigate the feasibility to integrate this information “live” (prospectively) during ischemic ventricular tachycardia ablation procedures.

## Introduction

Accurate characterization of the arrhythmic ventricular substrate is essential in preprocedural planning and procedural success of ventricular tachycardia (VT) catheter ablation. Cardiac imaging of left ventricular scar location and size have increased our arrhythmogenic substrate comprehension,^1^^,^^2^ which was linked to improved substrate-directed strategies for VT ablation.^3^, ^4^, ^5^, ^6^ Among the available imaging technologies for noninvasive scar delineation, multidetector computed tomography (CT) and contrast-enhanced magnetic resonance imaging are the most frequently used modalities for image integration during VT ablation procedures.^3^ However, there are major technical limitations, such as the artefacts created by defibrillator leads, limiting the accuracy of image integration with intraprocedural electroanatomic maps.^7^, ^8^, ^9^ A reliable, specific, and sensitive noninvasive method for delineating low-voltage areas is an unmet clinical need.

Despite being rarely used in integration with 3D electroanatomic maps during VT ablation, single-photon emission computed tomography (SPECT) imaging offers some important advantages over other imaging modalities. First, it is not affected by implanted cardiac devices/leads^10^; and second, it provides both anatomical information about areas of scar and physiological information with regard to myocardial perfusion and presence of ischemic zones.^4^ Recent technological advancements allow for the coupling of low-dose CT with SPECT technology, providing anatomical landmarks that can be used for co-registration with 3D mapping.^11^^,^^12^ However, a potential limitation of this imaging modality is its lower spatial resolution. Moreover, owing to a different set of challenges, SPECT data have yet to be integrated in the EnSite™ system (Abbott, St. Paul, MN) using impedance location. The aim of this study was to determine the feasibility and operating characteristics of integrating SPECT/CT images with electroanatomic voltage maps obtained with the EnSite Precision™ cardiac mapping system (Abbott) in patients undergoing scar-mediated VT ablation.

## Methods

### Study population

**Figure 1:**
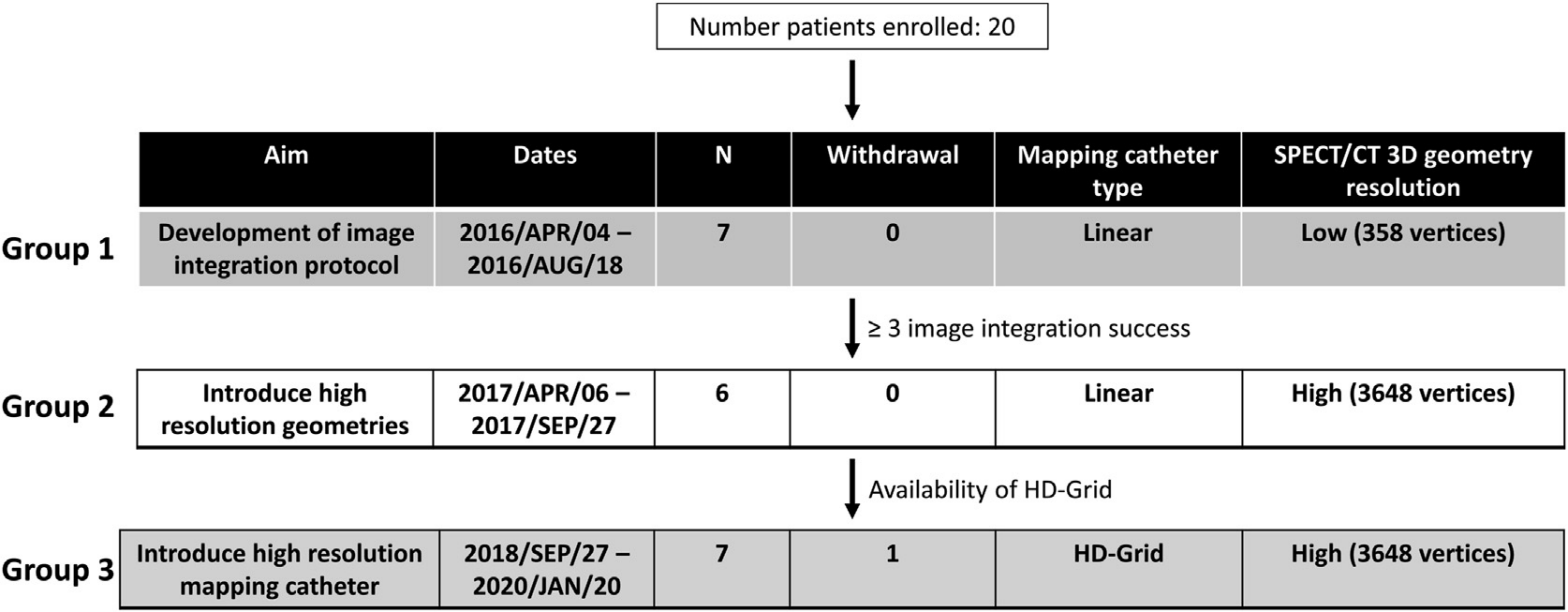
Impact of single-photon emission computed tomography/computed tomography (SPECT/CT) 3D geometry resolution. Experimental groups based on SPECT left ventricle 3D geometry resolution and type of mapping catheter used. HD = high-definition.

The study population consisted of 20 adult patients with implanted cardiac defibrillator and sustained scar-mediated left-ventricle VTs scheduled for an ablation procedure at the Montreal Heart Institute. Patients who were in good general condition with fewer ventricular arrhythmias were favored to enter the study. This may be reflected by a somewhat high proportion of previous inferior myocardial infarction. The enrollment period started on April 4, 2016, and lasted until January 20, 2020 (Figure 1). Patients with nonischemic substrate or in NYHA class IV were excluded.

The study protocol included the following 3 steps (Figure 1): (1) development of the image integration protocol (with lower resolution of SPECT/CT to facilitate data processing); (2) usage of higher-resolution of SPECT/CT in combination with a standard quadripolar mapping catheter; (3) usage of high resolution from both SPECT/CT (same as step 2) and 3D mapping. In step 3, 1 patient was withdrawn from the analysis owing to incomplete left ventricular mapping data acquisition. A 12-month hiatus occurred between steps 2 and 3, while awaiting approval of the HD Grid mapping catheter (Abbott) from the regulatory authorities in Canada. The protocol was approved by the Montreal Heart Institute’s Research and Ethics Committees, and adheres to the Helsinki Declaration Guidelines on Human Research. Written informed consent was obtained from the patients.

### SPECT/CT data acquisition

All subjects went through a rest nuclear imaging protocol prior to ablation to evaluate myocardial perfusion. Images were acquired using a Symbia^TM^ T6 SPECT-CT with SmartZoom collimators (Siemens Healthineers, Erlangen, Germany). A low-dose CT (30 mA / 130 kV) was acquired for attenuation correction and localization with a pitch of 1.0 and a slice thickness of 4 mm reconstructed every 2 mm. The SPECT data were acquired with a 16-frame electrocardiography-gated tomographic acquisition with 17 projections of 22 seconds using 2 detectors in step-and-shoot mode (matrix size was 128 × 128 pixels). SPECT slice thickness was 5 mm reconstructed every 5 mm.

### Myocardial perfusion segmentation

**Figure 2:**
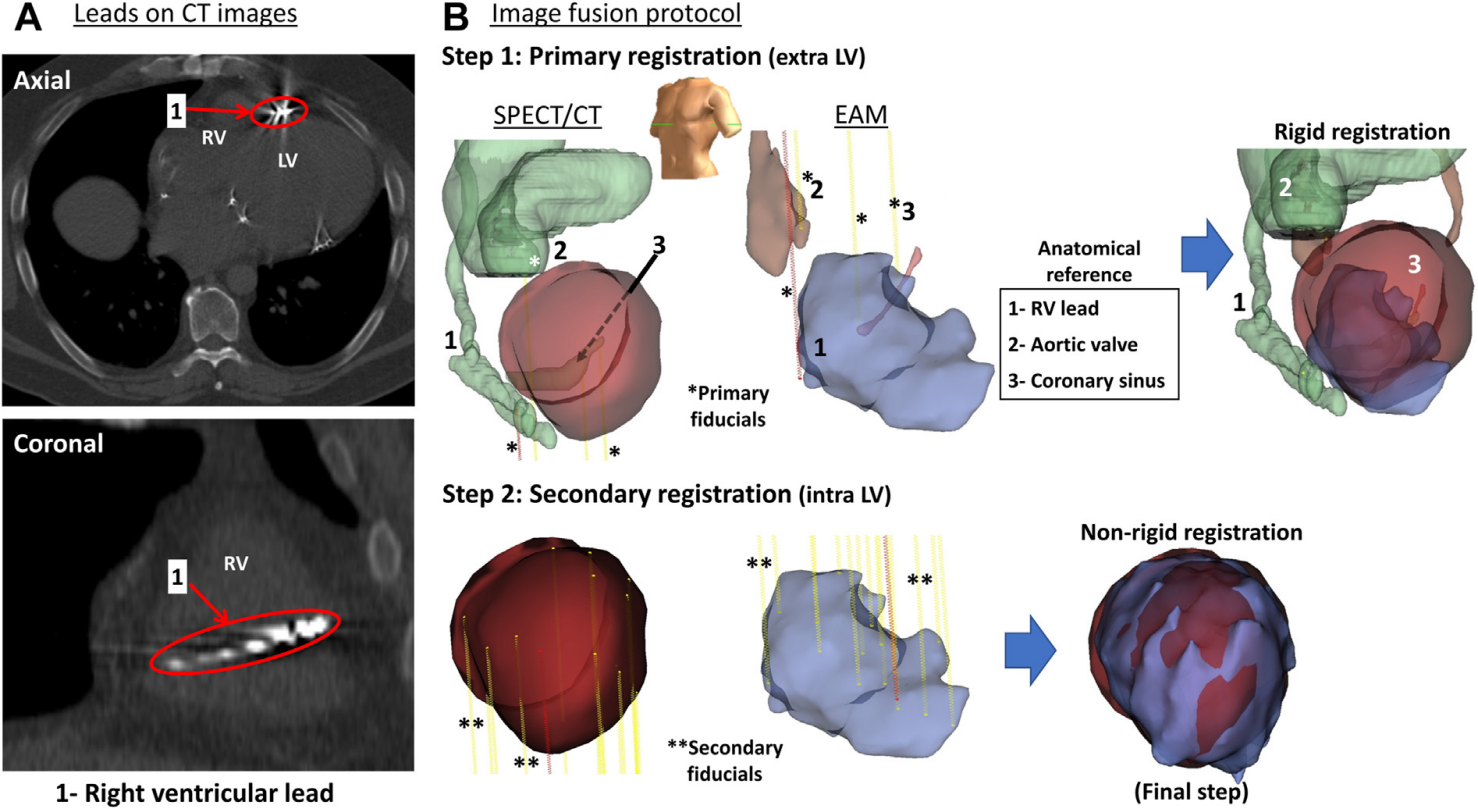
Computed tomography (CT) images and 3D geometry registration steps. **A:** Low-dose CT images (DICOM gray level images) shown in axial and coronary views, depicting device lead locations in the right ventricles. **B:** (1) Primary fiducial creation on fixed anatomical landmarks present on electroanatomical mapping (EAM) and single-photon emission computed tomography/computed tomography (SPECT/CT) geometries outside of the left ventricle (extra-LV) followed by a rigid registration placing the EAM–endocardial left ventricle (endoLV) within the SPECT-endoLV geometry. **(**2) Secondary fiducials creation on EAM-endoLV and corresponding location on SPECT-endoLV (intra-LV) for nonrigid registration. LV = left ventricle; RV = right ventricle.

The images were analyzed using the custom-made MHI_4_MPI segmentation^13^^,^^14^ software. The SPECT/CT endocardial surface (endocardial left ventricle geometry; SPECT-endoLV) was generated from the electrocardiography-gated end-diastolic images, with the 3D-Snake software, to match the cardiac cycle moment at which EnSite Precision geometries are obtained. The CT images were segmented using ITK-SNAP 2.2 (www.itksnap.org)^15^ to identify and trace the following anatomical landmarks: the coronary sinus, the pulmonary artery, the ascending aorta, and implanted device leads (Figure 2). From the imaging data acquired, the first 7 subjects (step 1) had 3D geometries with 358 vertices (low spatial resolution) while 3D geometries of subjects 8–20 had 3648 vertices (steps 2 and 3 high spatial resolution).

### Electroanatomical mapping and VT ablation

Endocardial geometry creation relied on impedance (EnSite Precision; Abbott Inc) and magnetic (MediGuide™ or EnSite Precision; Abbott Inc) points acquisition in sinus rhythm. In addition to geometry, voltage data were acquired, and electroanatomical mapping (EAM) was created by the EnSite Precision cardiac mapping system (EAM-endoLV). To improve accuracy, field scaling and respiratory compensation were applied. Bipolar electrograms were filtered at 30–300 Hz. Electrical identification of scar areas was done through bipolar EAM using a voltage amplitude–based criterion (with values <0.3 mV identifying “deep scar” and values >1.5 mV healthy myocardium). Fractionated signals identified either by the physician per-procedure using Jaïs and colleagues’ definition of local abnormal ventricular activation^16^ or through EnSite Precision fractionation maps (threshold = 3) were used to locate scar areas and arrhythmogenic substrate. Voltage data from EAM was our gold standard in the clinical decision to determine the ablation locations.

### Retrospective registration

The anatomical landmarks identification process was done in 2 steps: (1) intraprocedural location of SPECT/CT landmarks on the EnSite Precision geometry; and (2) postprocedure review of the LV cardiac chamber, along its long (basal, mid, and apical) and short (anterior, septal, and inferior) axes, with the physician to confirm the LV landmarks identification on the EnSite geometry. Anatomical landmarks identified included the right atrial lead (if present), the distal end of the right ventricular lead coil, the right ventricular apex, coronary sinus, aortic arch, aortic valve, pulmonary veins, mitral valve, and left ventricular apex.

Offline retrospective registration started with a rigid registration using 3 of the primary fiducial points mentioned above^17^ (Figure 2B, step1). Secondary fiducial points were established on the LV,^17^ orthogonally to each other, to insure a nonrigid registration with even distribution of its spatial effect (Figure 2B, step 2). A total of 12–15 fiducial points was needed to complete the registration process. Voltage data were then projected on SPECT-endoLV^18^ and the resulting map exported in the same Excel file containing the perfusion values.

### Data scoring method and statistics

**Figure 3:**
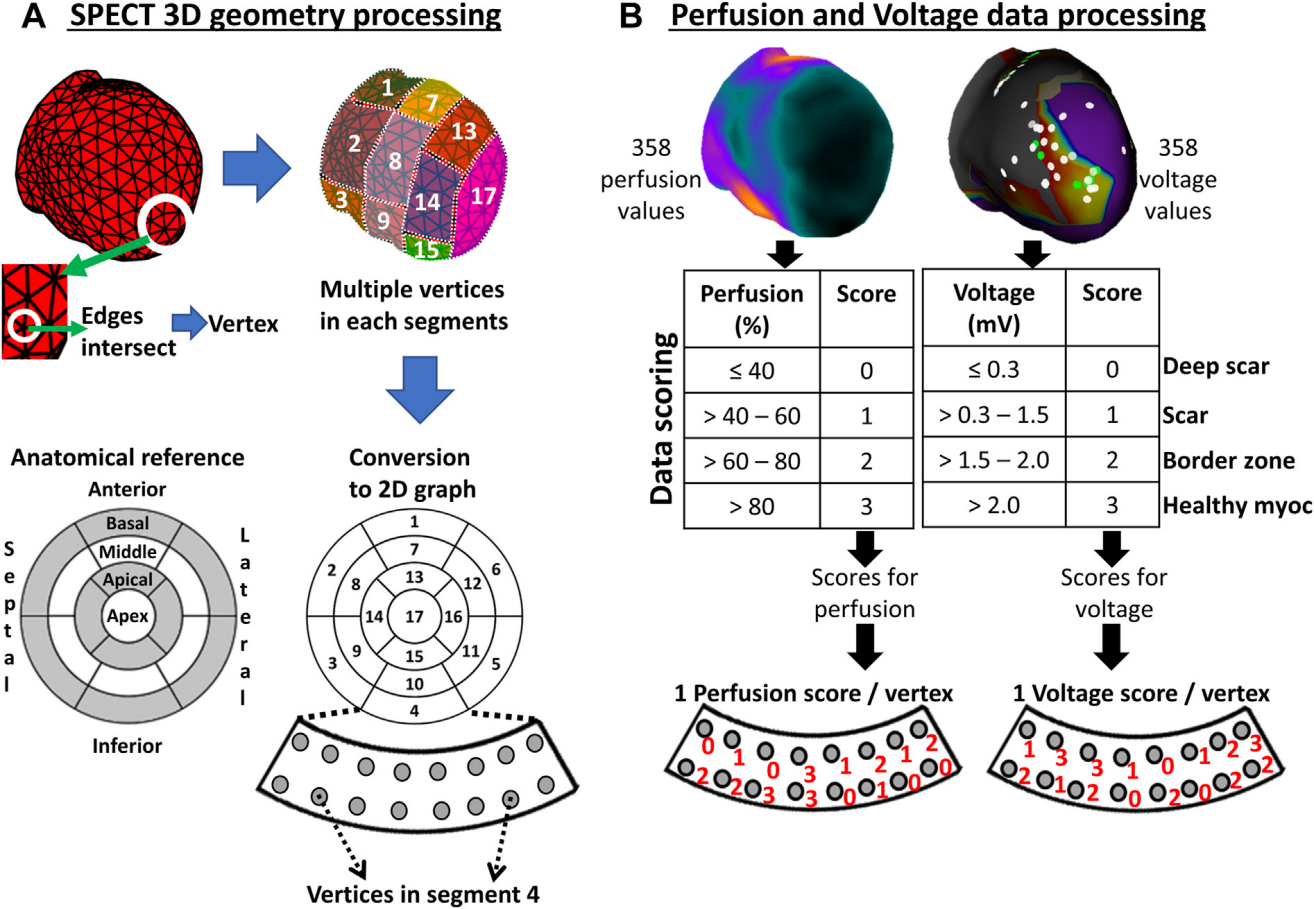
Data processing method. **A:** Edges intersect creates vertices on single-photon emission computed tomography–endocardial left ventricle (SPECT-endoLV); the latter is subdivided into 17 segments and converted in a 2-dimensional graph on which segments and associated vertices are transferred. **B:** Following fusion, voltage data are projected on SPECT-endoLV, allowing comparison with perfusion data. Lower panel depicts an example of the scoring result in the same segment for both data subsets. 2D = 2-dimensional; 3D = 3-dimensional; myoc = myocardial.

The SPECT-endoLV mesh consisted of faces, edges, and vertices (edges intersection on Figure 3A) distributed in 17 segments. A scoring system was developed in MATLAB® (version R2017b, MathWorks, Natick, MA) for direct comparison of semiquantitative perfusion values to voltage data (Figure 3B) and evaluation of registration accuracy. These scores reflected tissue viability in an ascending order (score of 3 = high viability). Segments without voltage data but known to be healthy based on perfusion data were assigned a score of 3. Perfusion and voltage scores were averaged by segment; when standard deviation was higher than the mean, segments were qualified as heterogeneous.

A receiver-operator curve (ROC) generated with SPSS-Statistics version 25 (IBM Corporation, Armonk, NY) was used to determine the score threshold value used for Viable/Not Viable segment classification. This first analysis led to voltage averaged score classification in 1 of 4 categories for each segment: (1) true positive (perfusion/voltage ≥ threshold); (2) true negative (perfusion/voltage < threshold); (3) false negative (perfusion ≥ threshold); (4) false positive (voltage ≥ threshold). Concordance, sensitivity, specificity, and positive and negative predictive values were derived from the above classification.

## Results

### Characteristics of study population and voltage mapping procedure

Twenty subjects (Table 1) underwent standard SPECT/CT imaging protocol followed by VT ablation; an average of 16 ± 17 days elapsed between the SPECT/CT imaging at rest and the ablation procedure. One patient from group 3 was withdrawn from the final analysis owing to incomplete EAM and limited ablation owing to the proximity of the arrhythmogenic substrate with the proximal atrioventricular conduction system (distal His / proximal left bundle). Overall, SPECT/CT imaging identified ischemic scar tissue in all subjects, distributed in the inferior (n = 15), in the anteroseptal (n = 2), or around the apical (n = 3) LV segments. The mean ablation procedure time was 244 ± 55 minutes. On average, 702 ± 280 (steps 1 and 2) and 1804 ± 433 (step 3) voltage points were collected over an estimated time range of 30–45 minutes. A total of 47 VT episodes were induced among the subjects (range: 0–4), all with isthmus localized in regions of scar by electroanatomic voltage mapping. The average time for an ablation procedure was 244 ± 55 minutes, during which an average of 42 ± 19 ablation lesions were created through delivery of a total of 4729 ± 2395 W (data available in 13 of 21 patients).

**Table 1:** Patients’ characteristics

Total number of patients	20
Male, n	19
Mean age (years) ± SD	67 ± 7
Mean LV ejection fraction, %	36 ± 11
Coronary artery disease, n	20
Previous myocardial infarction, n	18
Previous revascularization intervention, n (PCI, CABG)	12
Median number of VT episodes in past year (IQR)	2 (1.5, 3.5)

CABG = coronary artery bypass graft; IQR = interquartile range; LV = left ventricular; PCI = percutaneous coronary intervention; VT = ventricular tachycardia.

### Nuclear imaging and voltage mapping retrospective registration

**Figure 4:**
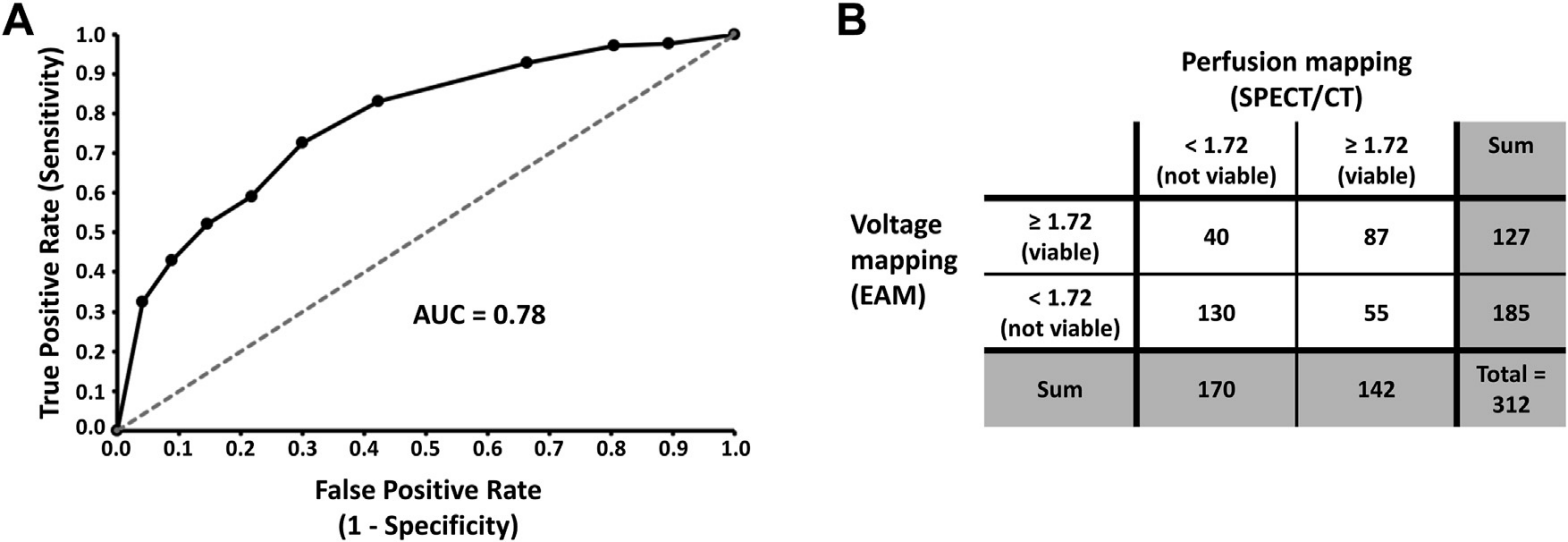
Evaluation of the Viable/Not Viable binary classification by voltage mapping compared to perfusion mapping (the gold standard). **A:** Receiver-operator curve translating voltage mapping’s ability to predict perfusion data when projected on the single-photon emission computed tomography/computed tomography (SPECT/CT) geometry. **B:** Summary of the true/false positive and negative following score classifications by voltage mapping. AUC = area under the curve; EAM = electroanatomical mapping.

A ROC analysis provided a score threshold of ≥1.72 to classify perfusion or voltage averaged scores as viable, with an area under the curve of 0.78 (Figure 4A). With this threshold, 87 of 312 segments (28%) were labelled as viable and 130 (42%) as not viable (total = 217 segments, or 70%) by both perfusion and voltage mapping technologies (Figure 4B). The concordance of the EAM segments with the perfusion segments was 70%. Four out of 19 subjects had a successful data match with a concordance superior to 80% and 8 additional subjects had a concordance between 70% and 80%. Voltage averaged scores overestimated the scar in 17.6% of all segments, conferring to our registration method a negative predictive value of 70.3%. Similarly, the average scores derived from voltage failed to identify 12.8% of all segments defined as not viable with SPECT, providing a positive predictive value of 68.5%. The averaged sensitivity, reflecting the pairing of the viable segments from perfusion and voltage mapping, was 61.3%. Correct matching of nonviable segments was more accurate, with an averaged specificity value of 76.5% when comparing voltage to perfusion mapping data. All heterogeneous segments were associated with segments characterized as not viable by perfusion or voltage or both.

### Impact of SPECT/CT 3D geometry resolution

**Figure 5:**
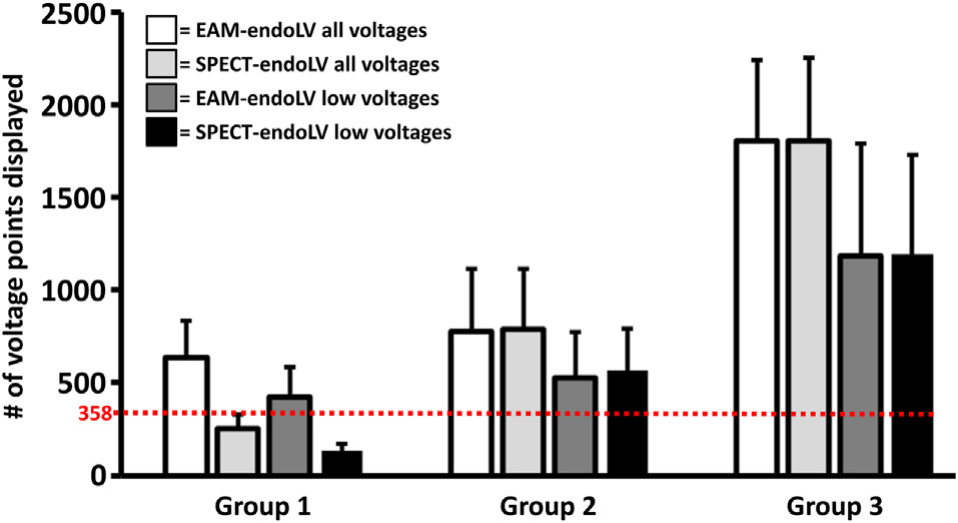
Impact of single-photon emission computed tomography (SPECT)/computed tomography 3D geometry resolution. Number of voltage points acquired; all voltages included or focusing on low voltages (<1.5 mV) only, displayed on electroanatomical mapping–endocardial left ventricle (EAM-endoLV) and SPECT-endoLV with respective spatial resolution; red dotted line indicates potential impact of SPECT-endoLV lowest spatial resolution on groups 2 and 3.

The 19 subjects were distributed in 3 steps based on SPECT LV 3D geometry resolution and type of mapping catheter used (Figure 1). The initial voltage mapping on EAM-endoLV led to a total of 639 ± 197 points used in group 1, 776 ± 338 points used in group 2, and 1804 ± 433 points used in group 3 (Figure 5). Following image integration, EAM-endoLV voltage data were displayed on SPECT-endoLV (348 vertices) and led to a 60% ± 10% decrease of the voltage points used for subjects in step 1. No voltage data loss occurred upon projection on high-resolution SPECT-endoLV (3648 vertices) for patients in steps 2 or 3. The decrease of EAM-endoLV bipolar endocardial voltage lower than 1.5 mV displayed on low-resolution SPECT-endoLV rose to 67% ± 10%. Similarly to all voltage data projection, no data loss occurred in steps 2 and 3, not even when using a high-resolution mapping catheter in the last group.

## Discussion

Image integration is a complex, multistep process in which the final goal is to provide additional, complementary to EAM, information on the cardiac substrate for the physician’s therapeutic roadmap. Image integration generated a positive impact on clinical outcomes in LV substrate ablation procedures.^6^ Each of the 4 steps involved in the integration of an external 3D geometry in the EnSite Precision environment (specific system reference, 3D geometry, etc) introduces its own intrinsic error. In step 1, the cardiac cycle in which the images are acquired is important because EAM produces 3D geometries that better correspond to diastole. Image segmentation accuracy (step 2), used to build 3D geometries, can be affected by reflection artefacts introduced by leads in the cardiac chamber of interest. Step 3 is a crucial step of image integration, since it deals with the spatial link creation between the imaging and EAM geometries and associated data^1^ through registration with fiducial points. Its accuracy is directly linked to the presence of anatomical landmarks identifiable on both geometries. Finally, in step 4, the calculations for the imaging/EAM geometries fusion use the fiducial points and will allow tool localization and navigation in the 3D geometries. Thus, accurate therapeutic roadmaps are directly linked to high registration and fusion accuracy.

Multidetector CT or contrast-enhanced magnetic resonance imaging typically provide 3D geometries with the best spatial resolution, but presence of intracardiac leads creates artefacts of higher intensities compared to SPECT/CT technology. Owing to its lower cost and extensive clinical validation, SPECT is widely used for evaluation of myocardial perfusion and is minimally impacted by presence of ventricular leads. However, a potential limitation of this imaging modality is its lower spatial resolution, limiting the accuracy to accurately locate proarrhythmic isthmus, for instance. The present study proposed an accessible SPECT/CT protocol for 3D image integration, suitable for patients with implanted devices and intracardiac leads. The experimental groups in this study allowed our team to optimize each step of image integration mentioned above, with a special emphasis on registration (step 3). The fiducial point–based registration method, most commonly used for 3D geometry integration,^17^^,^^19^ benefited from extra-LV rigid structures segmented from CT images in our protocol to compensate for the lack of accurate anatomical landmarks (eg, papillary muscle locations; Figure 2A) on SPECT-endoLV. Segmentation of rigid structures has been shown to help reduce rotational errors when integrating SPECT/CT and EAM LV geometries.^20^ Accuracy of cardiac lead segmentation was impacted by cardiac motion, a frequent source of error,^17^ and minor lead artefacts on CT images, resulting in 3D cardiac lead geometries with some inaccuracies.

With this protocol, we were able to demonstrate the feasibility of retrospective SPECT/CT 3D geometry integration in the EnSite Precision cardiac mapping system for patients with implanted devices. Such integration provided preprocedural data on LV substrate, including scar (location, size), in complement to the per-procedural voltage maps. This is similar to previous publications that indicated that SPECT data could be (1) used for scar identification and (2) registered with a geometry from a cardiac mapping system when using a magnetic-based navigation system.^10^ The methodology developed in the present study addressed the different challenges posed by an impedance-based navigation system when integrating SPECT/CT data.^10^ Specifically, we were able to accurately proceed to cardiac lead segmentation feasibility in low-resolution CT images and to project EnSite Precision voltage mapping data on SPECT/CT geometry. The scoring system developed to compare the semiquantitative (SPECT/CT) to the quantitative (EAM) mapping values demonstrated a good concordance of the scar location between the 2 types of data. A ROC analysis of the scores revealed 1.72 to be the most clinically relevant threshold for optimal classification of the “Not Viable” tissue, targeted by the ablation therapy, with an area under the curve of 0.78.

Three-dimensional spatial resolution was also investigated for imaging and EAM 3D geometries in this study. As expected, accuracy was improved with technology amelioration. The additional points acquired by high-resolution mapping catheters leads to improved geometry definition and detailed voltage maps. Publications comparing high-resolution catheter to point-by-point mapping with linear catheters reported LV substrate voltage maps with a density of points used ranging between 1500 and more than 3000 with high-resolution catheter.^21^, ^22^, ^23^ Likewise, SPECT/CT 3D geometries are composed of vertices, ie, coordinate in space, that will define the geometry spatial resolution, including the anatomical landmarks, and be associated to physiological values (tissue perfusion, voltage, etc) to display different maps. Anatomically, well-defined imaging and EAM geometries will provide exhaustive anatomical landmarks for an increased registration accuracy. Physiologically, high-spatial-resolution geometries will translate into precise maps, offering an improved comprehension of the LV substrate. The accuracy of voltage maps to describe LV substrate can be impacted by different parameters, such as the orientation of the electrode pair and catheter contact with the LV wall. Preprocedural availability of imaging data (geometry and perfusion maps) is one of the tools available to help physicians assess the validity of the electrical map in acquisition. However, as depicted in step 4 of image integration (geometry fusion) also led to voltage data projection on SPECT-endoLV. As shown in this study, a voltage map projection on low-resolution SPECT-endoLV (small number of vertices), as in group 1 in the present study, could have a critical impact on substrate comprehension, notably when mapping specific smaller scar regions to identify conduction channels. Similarly, a low-resolution voltage map projected on a high-resolution imaging geometry can lead to misinterpretation of the substrate. Thus, the spatial discrepancy between an imaging and EAM geometries will dictate the quality of the postfusion maps used to establish the physician’s therapeutic roadmap.

### Study challenges and future development

The enrollment timeline associated with this pioneer study was one of the main challenges faced, owing to the unpredictable availability of subjects, the constant need to improve the workflow between nuclear imaging and electrophysiology, and the decision to wait for commercial availability of the Advisor HD Grid Mapping Catheter, Sensor Enabled in 2018.

Technological limitations linked to the data included the following: (1) perfusion semiquantitative scale, representing an average through the LV thickness, which necessitated use of a nonparametric scale to determine the registration accuracy; (2) the use of the impedance field for tool localization and navigation, which, owing to patient torso impedance inhomogeneities, prevented the direct computation of registration errors; and (3) missing voltage data in segments unrelated to the VTs or inaccessible to operator catheter. Retrospective nonrigid registration can alter the EnSite geometry and its voltage map, leading to data projection at a different location than its intended site on SPECT-endoLV. This could have been improved if full registration had been performed during the procedure, allowing for more complete and more accurate anatomy collection. In combination with pre-existing perfusion/voltage map discrepancy owing to the voltage map’s focus on the LV endocardial surface, the ablation catheter for mapping in steps 1 and 2 may have mitigated registration accuracy with SPECT/CT, the latter providing “transmural” information, with no capacity to discriminate between endocardial, midmyocardial, and epicardial components. Among SPECT/CT imaging limitations reported,^10^ its spatial resolution (≈12 mm) resulted in endocardial LV geometries without anatomical features (no papillary muscle or chordae) to help the intra-LV registration process. This will represent a challenge at the time of identifying accurately the areas of interest for ablation, as VT circuits are not necessarily limited to the endocardial surface.

Also, the version of EnSite Precision used during the study was based on impedance, which is known to be less accurate than magnetic systems. Despite this limitation, we were able to get a 70% concordance between the EAM and SPECT-CT data. We are confident that this concordance could be improved with the latest EnSite system.

Finally, the combination of SPECT/CT and EAM data collected by electrophysiologists led to a significant increase of the dataset size. Given that SPECT/CT imaging includes a growing number of radioactive tracers to map multiple parameters like impaired metabolic function or abnormal sympathetic innervation,^4^^,^^24^ the resulting size of the datasets will continue to grow, especially when combined with electrical data. To avoid overload, a need to develop a methodology to simplify/summarize the information related to the substrate may become essential. The methodology developed in this study was tailored to the ischemic cardiomyopathy patient population, given the higher complexity of nonischemic cardiomyopathy and limited spatial resolution of the SPECT/CT imaging modality, this methodology would need further optimization before being used in nonischemic cardiomyopathy.

## Conclusion

Concordance has been shown between SPECT/CT and EAM data in VT scar identification. Low-dose CT images were not impaired by presence of cardiac lead, allowing their segmentation and utilization in the registration process. Data fusion involving multiple modalities (ie, imaging and EAM) allow capitalization of preprocedural knowledge and may provide increased insight into arrhythmogenic substrate comprehension. Following these preliminary results, the value of EAM-SPECT/CT integration will be evaluated in further analysis with regard to the VTs that were induced during the ablation procedures. This should validate our methodology and lead to prospective evaluation in a new series of ischemic VT ablation procedures. To see if this would result in improved procedural outcomes, larger randomized controlled studies will be needed.

## Acknowledgments

In the present study, the software version used for retrospective 3D image integration was not commercially available at the time of data acquisition and analysis. This software is property of Abbott Medical Inc.

The authors are grateful for all who were involved from the Montreal Heart Institute, especially the staff in the EP laboratories who were extremely supportive and patient during those long studies. We also thank Ms Antoinette Paolitto for her valuable secretarial support during the preparation and submission of the present manuscript. Finally, we also would like to express our gratitude to all the research team from Abbott Medical; without their support this study would not have been possible.
